# Characterization of the Apoptotic Response Induced by the Cyanine Dye D112: A Potentially Selective Anti-Cancer Compound

**DOI:** 10.1371/journal.pone.0125381

**Published:** 2015-04-30

**Authors:** Ning Yang, Paul Gilman, Razmik Mirzayans, Xuejun Sun, Nicolas Touret, Michael Weinfeld, Ing Swie Goping

**Affiliations:** 1 Department of Biochemistry, University of Alberta, Edmonton, Alberta, Canada; 2 Independent Consultant, Retired from Research Laboratories, Eastman Kodak Company. Rochester, New York, United States of America; 3 Department of Oncology, University of Alberta, Edmonton, Alberta, Canada; Rutgers University, UNITED STATES

## Abstract

Chemotherapeutic drugs that are used in anti-cancer treatments often cause the death of both cancerous and noncancerous cells. This non-selective toxicity is the root cause of untoward side effects that limits the effectiveness of therapy. In order to improve chemotherapeutic options for cancer patients, there is a need to identify novel compounds with higher discrimination for cancer cells. In the past, methine dyes that increase the sensitivity of photographic emulsions have been investigated for anti-cancer properties. In the 1970's, Kodak Laboratories initiated a screen of approximately 7000 dye structural variants for selective toxicity. Among these, D112 was identified as a promising compound with elevated toxicity against a colon cancer cell line in comparison to a non-transformed cell line. Despite these results changing industry priorities led to a halt in further studies on D112. We decided to revive investigations on D112 and have further characterized D112-induced cellular toxicity. We identified that in response to D112 treatment, the T-cell leukemia cell line Jurkat showed caspase activation, mitochondrial depolarization, and phosphatidylserine externalization, all of which are hallmarks of apoptosis. Chemical inhibition of caspase enzymatic activity and blockade of the mitochondrial pathway through Bcl-2 expression inhibited D112-induced apoptosis. At lower concentrations, D112 induced growth arrest. To gain insight into the molecular mechanism of D112 induced mitochondrial dysfunction, we analyzed the intracellular localization of D112, and found that D112 associated with mitochondria. Interestingly, in the cell lines that we tested, D112 showed increased toxicity toward transformed versus non-transformed cells. Results from this work identify D112 as a potentially interesting molecule warranting further investigation.

## Introduction

Programmed cell death, or apoptosis, is vital for proper development and homeostasis in virtually all tissues [1]. Two well-studied signaling processes termed the intrinsic and extrinsic pathways represent the major mechanisms of apoptosis [2]. Extrinsic apoptosis involves extracellular ligand-mediated activation of plasma membrane-localized death receptors, while intrinsic apoptosis is initiated by intracellular stresses, such as hypoxia, DNA damage, oxidative stress, and anti-cancer therapies [3]. These apoptotic stimuli lead to mitochondrial membrane permeabilization [4] and the release of apoptotic factors, such as cytochrome c [5], from the mitochondrial inter-membrane space. The intrinsic pathway is tightly regulated by the Bcl-2 family of proteins [6, 7] that comprise anti-apoptotic (such as Bcl-2, Bcl-XL, Bcl-w) and pro-apoptotic proteins (such as Bax, Bak, Bid, Bad, Bim) [8]. Pro-apoptotic proteins stimulate formation of the Bax/Bak pore [9] or regulate the existing mitochondrial permeability transition pore [10] to release mitochondrial apoptosis-inducing proteins. On the other hand, anti-apoptotic proteins, such as Bcl-2 and Bcl-XL [11, 12], inhibit mitochondrial dysfunction, thus blocking apoptosis. When pro-apoptotic Bcl-2 proteins prevail, the released cytochrome c stimulates formation of an oligomeric structure named the ‘apoptosome’ including apoptotic protease activating factor-1 and initiator caspase, caspase-9. This structure triggers the caspase cascade by activating downstream effector caspases such as caspase 3.

Apoptosis is widely blocked in cancer. Cancer is the second most common cause of death, accounting for nearly 1 in every 4 deaths in North America [13, 14]. Apoptosis deregulation often manifests as clinical anticancer treatment resistance. Additionally, off-target damage to non-cancerous tissue is a major toxicity to many drug regimens. Hence identifying drugs that more selectively target cancer cells and spare healthy tissue is the focus of many drug discovery initiatives.

**Fig 1:**
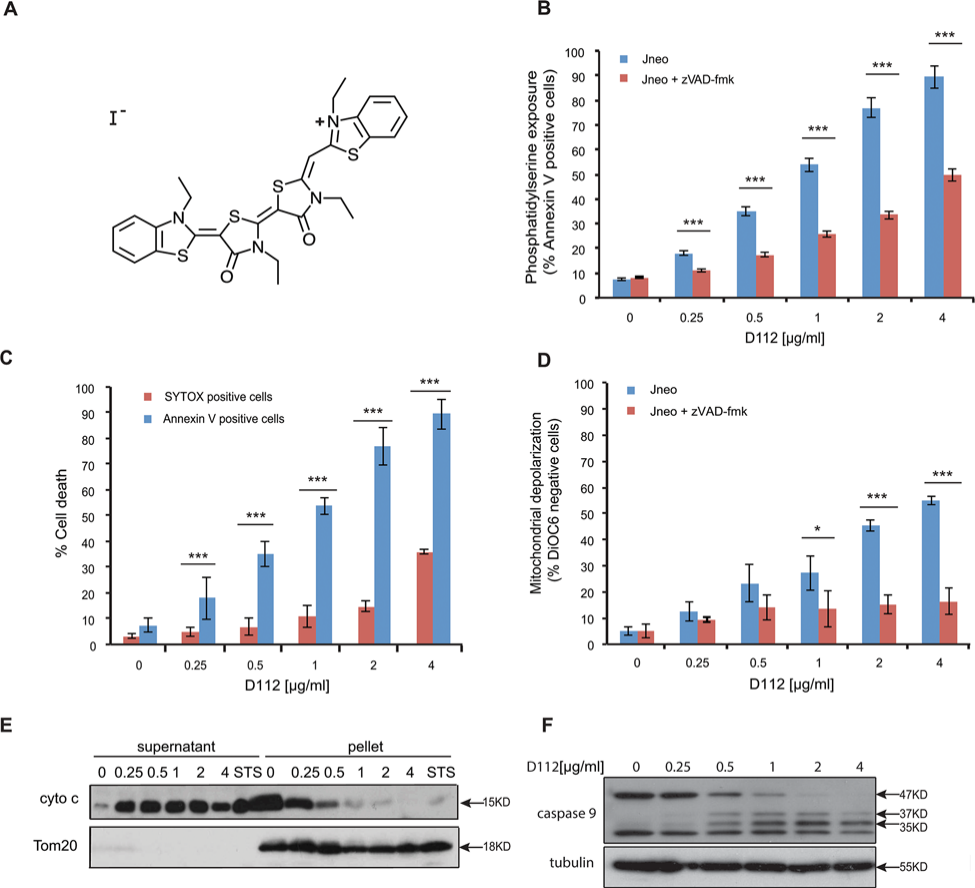
D112 induces apoptosis in Jurkat cells. (A) D112 structure. (B) Jneo cells were treated with the indicated concentrations of D112 for 24 h in the presence or absence of the pan-caspase inhibitor zVAD-fmk (20 μM). Phosphatidylserine exposure indicated by Alexa Fluor 647-annexin V positive cells is shown as a percent of total cells as determined by flow cytometry. Mean ± SD of three independent experiments performed in triplicate are shown, *P<0.05, **P<0.01, ***P<0.001. (C) Necrosis detection. Cells were treated as above and were incubated with SYTOX Green stain or Alexa Fluor 647-annexin V. Mean ± SD of three independent experiments performed in triplicate are shown, *P<0.05, **P<0.01, ***P<0.001. (D) Mitochondria dysfunction. Cells were treated as above and incubated with DiOC_6_. Loss of electrochemical potential was determined by loss of fluorescence in the FL-1 channel as measured by flow cytometry. Mean ± SD of three independent experiments performed in triplicate are shown, *P<0.05, **P<0.01, ***P<0.001. (E) Cytochrome c release. Cells were incubated with D112 at indicated concentrations for 24 h and then harvested and fractionated. Supernatant and pellet were loaded separately and subjected to western blot analysis. The experiment was performed independently three times and a representative blot is shown. (F) Caspase 9 cleavage. Cells were incubated for 24 h with increasing concentrations of D112 as indicated. Whole cell lysates were subjected to western blot analysis with the indicated antibodies. The experiment was performed independently three times and a representative blot is shown.

Our studies focused on elucidating the molecular mechanism of toxicity of a cyanine molecule, D112, and evaluating its cancer cell selectivity. D112 was identified by the Eastman Kodak Company through a cancer drug-screening program initiated at the Dana Farber Institute in the1970’s [15]. Initial observations deduced that the electrochemical reduction potential of a dye influenced the ability of the dye to inhibit the growth of healthy sea urchin eggs [16]. Using this rationale, the Kodak Laboratory employed electrochemical reduction potential to select drugs for screening of anti-cancer properties. Approximately 7000 dye structural variants were tested and D112 (Fig 1A), emerged as a lead compound with higher cytotoxic activity against a cancer cell line versus a non-transformed cell line [15]. Specifically, the IC50 of D112 to the normal monkey kidney epithelial cell line CV-1 was 9 μg/ml, compared to 0.01 μg/ml to the human colon cancer cell line CX-1, thus achieving a killing ratio (IC50 CV-1/ IC50 CX-1) of 900 [15]. Furthermore, D112 showed enhanced selectivity compared to the widely used clinical chemotherapeutic agent, adriamycin. Despite these promising results, due to changes in industry priorities, investigations into D112 were not further pursued.

Currently, there is still a pressing need to identify new drugs that can discriminate cancer cells from normal cells, so we decided to revive studies on D112. Our study investigated the cytotoxicity of D112 in cancer cells, characterized D112-induced apoptosis, and further explored its possible selective activity against cancer in comparison to normal cells.

## Results

### D112 induces apoptosis in Jurkat cells

To investigate whether D112 can induce apoptosis, we treated Jurkat cells (a human T-cell leukemia sub-line, named Jneo [17]) with D112 at increasing concentrations for 24 h and measured the apoptotic hallmarks of phosphatidylserine externalization, mitochondrial depolarization, and caspase activation. We first assessed cell death with the apoptotic marker of phosphatidylserine (PS) exposure. PS is normally restricted to the inner leaflet of the plasma membrane bilayer, and is externalized in response to caspase activation [18]. To measure whether D112 induced PS exposure, we harvested cells after D112 treatment and measured fluorescent-annexin V binding by flow cytometry. The addition of D112 increased Annexin V positive cells in a dose-dependent manner (Fig 1B). To determine whether PS exposure was caspase-dependent, we co-incubated cells with the pan-caspase inhibitor zVAD-fmk and established that D112-mediated PS externalization was caspase-dependent. Of note, at higher concentrations of D112, a significant amount of PS exposure was observed in the presence of zVAD-fmk, relative to non-D112-treated cells. These results suggested that D112 either induced an alternative caspase-independent necrotic pathway, or that secondary necrosis (in vitro membrane damage) accounted for Annexin V positivity at higher concentrations of D112. To distinguish between these two possibilities, we measured plasma membrane disruption through uptake of the vital DNA-binding dye, SYTOX green (Fig 1C). Membrane damage was only evident at D112 concentrations that were higher than the amount required to detect PS exposure indicating that apoptosis and not necrosis was the initiating event. To directly measure secondary necrosis, we simultaneously labeled D112-treated cells with Alexa Fluor 647 Annexin V and SYTOX green in a time course experiment (S1 and S2 Figs). SYTOX green-only positive cells were undetectable. Instead, cells became Annexin V positive first and only at later time points became double positive for Annexin V and SYTOX green. Moreover, addition of the pan caspase inhibitor zVAD-fmk, was not associated with production of SYTOX green positive cells indicating that zVAD-fmk and D112 did not induce primary necrosis as has been demonstrated in other systems [19].

Since mitochondria are central regulators of the apoptotic pathway, we also examined mitochondrial electrochemical depolarization in response to D112 treatment. Mitochondrial depolarization was measured by the loss of fluorescence of the potentiometric dye DiOC_6_ (3) (3,3'-Dihexyloxacarbocyanine Iodide) (Fig 1D). Our study showed that the proportion of DiOC_6_ negative cells increased in response to D112 treatment. Furthermore, incubation with zVAD-fmk inhibited D112-induced mitochondrial depolarization, indicating that mitochondrial electrochemical potential loss was downstream of caspase activation. We next tested whether D112 induced the release of cytochrome c from the mitochondria. We incubated cells with increasing concentrations of D112 and fractionated cell components into heavy membrane and cytosolic fractions (Fig 1E). D112 induced the release of cytochrome c from the mitochondrial pellet fraction into the cytosolic supernatant fraction. Mitochondrial involvement and release of cytochrome c suggested that D112 would induce activation of the initiator caspase 9. In support of this, we observed activation-associated cleavage of caspase 9 in response to D112 incubation (Fig 1F). These results are consistent with a model whereby D112 induces cell death via the mitochondrial intrinsic apoptosis pathway.

**Fig 2:**
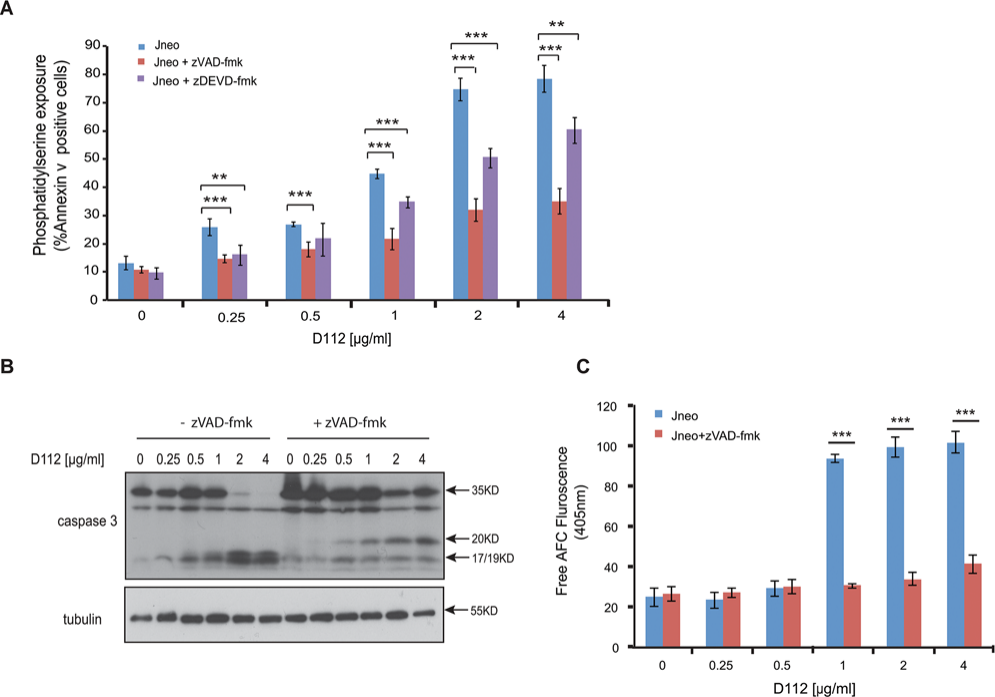
D112 induces caspase activation in Jurkat cells. (A) Phosphatidylserine exposure. Jneo cells were treated with indicated concentrations of D112 for 24 h in the presence or absence of the caspase 3 inhibitor z-DEVD-fmk (20 μM), stained as indicated and analyzed by flow cytometry. Mean ± SD of three independent experiments performed in triplicate are shown, *P<0.05, **P<0.01, ***P<0.001. (B) Caspase cleavage. Cells were incubated for 24 h with increasing concentrations of D112 as indicated. Whole cell lysates were subjected to western blot analysis with the indicated antibodies. The experiment was performed independently three times and a representative blot is shown. (C) Caspase 3 enzymatic activation. Jneo cells were treated with D112 in the presence or absence of z-VAD-fmk (20 uM) for 24 h prior to lysis. Cell lysates were incubated with the caspase 3 specific fluorometric substrate, Ac-DEVD-AFC, for 1 h and caspase 3 activity was measured at 405 nm. Mean ± SD of three independent experiments performed in triplicate are shown, *P<0.05, **P<0.01, ***P<0.001.

Caspase 9 activation classically leads to executioner caspase 3 activation thus we tested the requirement for, caspase 3. Incubation with the caspase 3-selective inhibitor zDEVD-fmk significantly reduced D112 induced apoptosis (Fig 2A). We then assessed the activation profile for caspase 3. Caspases are present as inactive pro-enzymes and are activated by proteolytic cleavage [20]. In response to increasing concentrations of D112, we observed the cleavage of pro-caspase 3 from 35 KD to the subunits p17/19 (Fig 2B). Addition of zVAD-fmk inhibited the D112-dependent production of p17/19 and instead produced an intermediate p20 fragment. To assess whether the p20 subunit was associated with enzymatic activity, we incubated cell lysates with the caspase 3 fluorogenic substrate A_C_-DEVD-AFC and measured fluorescence of free AFC. D112-treated cells harbored significantly increased caspase 3 enzymatic activity, while cells that had also been co-incubated with zVAD-fmk had background levels of enzymatic activity (Fig 2C). These results indicated that D112 induced the cleavage of caspase 3 to p20. However, the p20 product was not catalytically active, with subsequent caspase activity generated by the production of active p19/17 subunits of caspase 3.

We also assessed the BH3-only class of Bcl-2 family proteins to query upstream transducers of D112 toxicity [21, 22] (S3 Fig). Bid was cleaved in D112 treated cells, but this event was inhibited by zVAD-fmk, indicating that Bid cleavage potentially acted as an amplification mechanism of D112 toxicity, downstream of caspase activation. Puma, Bik, Noxa, and Bim levels did not increase in response to D112.

All together, our results indicated that D112 induced apoptosis as evidenced by caspase activation. Caspase activity induced both PS externalization and mitochondrial dysfunction. Therefore, we next decided to assess the functional requirement of mitochondrial dysfunction for D112-induced cytotoxicity.

### Anti-apoptotic Bcl-2 blocks D112-induced cell death

**Fig 3:**
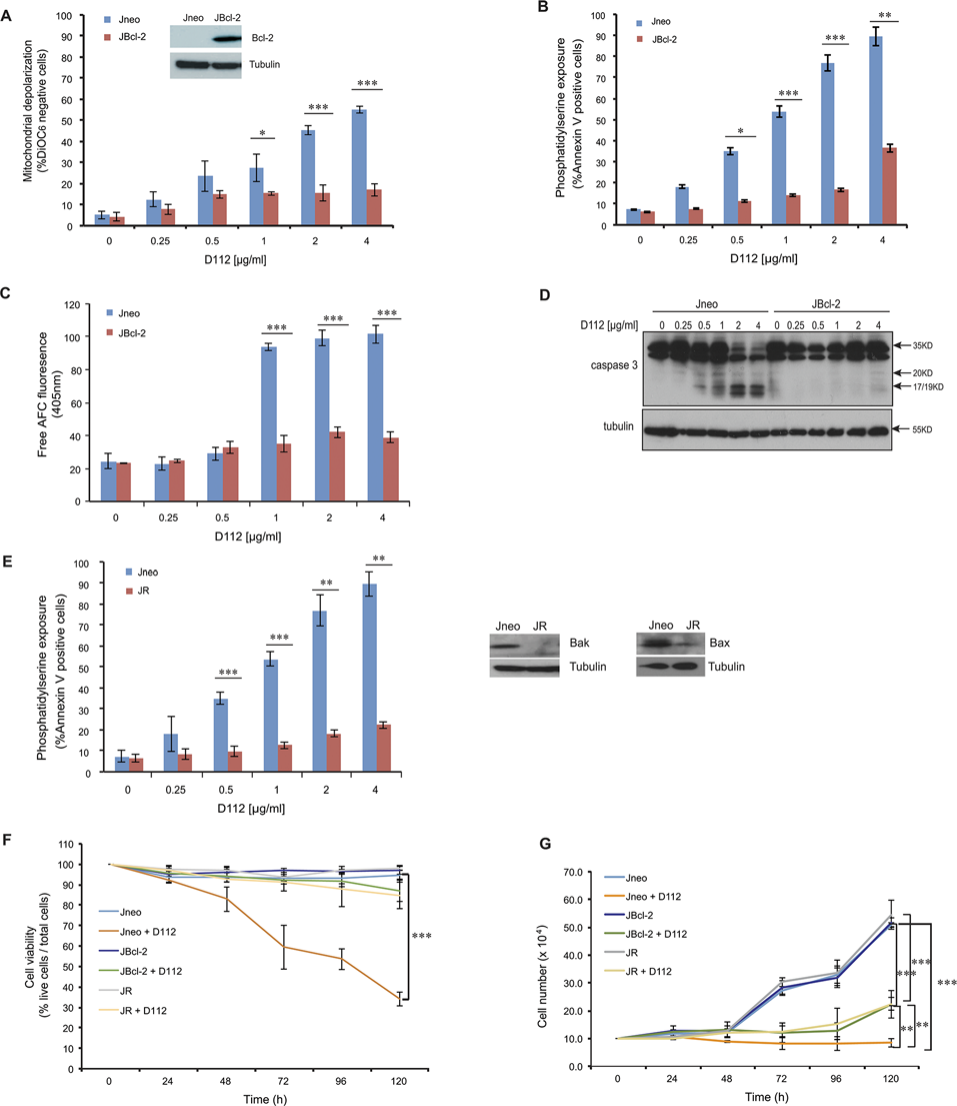
D112-induced apoptosis is blocked by Bcl-2. Jneo and JBcl-2 cells were treated with the indicated amounts of D112 for 24 h. Apoptotic cell death was measured by (A) mitochondrial dysfunction, (B) phosphatidylserine exposure, (C) caspase 3 enzymatic activation, and (D) caspase cleavage. All of these apoptotic hallmarks were quantitated as described previously. (E) Bax/Bak expression is required for D112-induced cell death. Jneo and JR cells were treated with indicated concentrations of D112 for 24 h and phosphatidylserine positivity was recorded as done previously. (F) Inhibition of the apoptosis pathway protects cell from D112-induced cell death. Cells were treated with 62.5 ng/ml D112 for 24 h. Cells then were incubated in fresh medium and counted daily. Cell survival was detected by trypan blue staining. (G) Cell proliferation is inhibited by D112. Cells were treated with 62.5 ng/ml D112 for 24 h. Cells then were incubated in fresh medium and total live cell number was counted. The expression of Bcl-2, Bax and Bak were verified in western blot insets in A and E. In all experiments, the mean ± SD of three independent experiments performed in triplicate are shown, *P<0.05, **P<0.01, ***P<0.001.

To explore the role of mitochondrial dysfunction in D112-induced apoptosis, we examined D112 sensitivity in Jurkat cells ectopically expressing Bcl-2 (JBcl-2). We observed that both D112-induced mitochondrial depolarization (Fig 3A) and PS exposure (Fig 3B) were significantly inhibited in JBcl-2 cells. Since caspase activation was critically important for PS exposure and mitochondrial depolarization, we directly measured enzymatic activity. As expected, we found that D112-treated JBcl-2 cells had background levels of caspase 3 activity (Fig 3C). Further, analysis of caspase cleavage patterns demonstrated that JBcl-2 cells showed no production of active subunits of caspase 3 (Fig 3D). These results confirmed that the mitochondrial pathway was critical to induce caspase activation in response to D112.

As a complementary approach, the involvement of mitochondrial dysfunction in D112-induced apoptosis was also confirmed by examining PS exposure in a sub-line of Jurkat cells (named JR) with diminished levels of Bax and Bak proteins [23, 24]. In response to apoptotic stimuli, pro-apoptotic proteins Bax and Bak form pores in the mitochondrial outer membrane, causing the release of mitochondrial proteins inducing apoptosis [9, 25]. Given that Bcl-2 blocked D112-induced cytotoxicity, we reasoned that loss-of-function of the pro-apoptotic proteins Bax and Bak would have a similar effect. Indeed in JR cells, PS exposure was not induced by D112, indicating that Bax/Bak was required for D112-induced cell death (Fig 3E). D112 did not appear to require the Permeability Transition Pore, as cyclosporin A did not inhibit D112-induced cell death (S4 Fig). These results confirmed the crucial role of mitochondrial dysfunction in D112-induced apoptosis and more specifically, that D112 induced a Bax/Bak-dependent mitochondrial apoptotic pathway triggering caspase activation.

To determine whether the extrinsic pathway contributed to D112-induced apoptosis, we examined caspase 8 activation by western blotting using a caspase 8 cleavage-specific antibody (S5 Fig). D112 induced caspase 8 cleavage, however, this was blocked by Bcl-2, indicating that caspase 8 activation was downstream of mitochondrial dysfunction and not dependent on extrinsic apoptotic mediators. To assess whether caspase 8 activation contributed to D112-induced apoptosis, we assayed Jurkat cells that stably expressed the rabbitpox virus-encoded caspase inhibitor SPI-2 that are resistant to extrinsic-mediated apoptosis [26]. These cells were equally sensitive to D112 treatment as control cells indicating that caspase 8 is dispensable for D112-mediated apoptosis.

We next assessed the effects of D112 treatment on cell viability. A four-fold lower dose than was used in the previous apoptosis assays showed a gradual decrease in viability of Jneo cells over 5 days, as determined by trypan blue staining. In contrast, both the presence of Bcl-2 or the depletion of Bax/Bak inhibited D112-induced cell death (Fig 3F). To assess whether D112 also affected proliferation, we counted the total number of live cells daily. D112 inhibited the proliferation of the JBcl-2 and JR cells, with significant difference to Jneo cells by day 5 (Fig 3G). Therefore, low doses of D112 induced apoptosis dependent on mitochondrial dysfunction and arrested cell proliferation.

### D112 co-localizes with mitochondria

**Fig 4:**
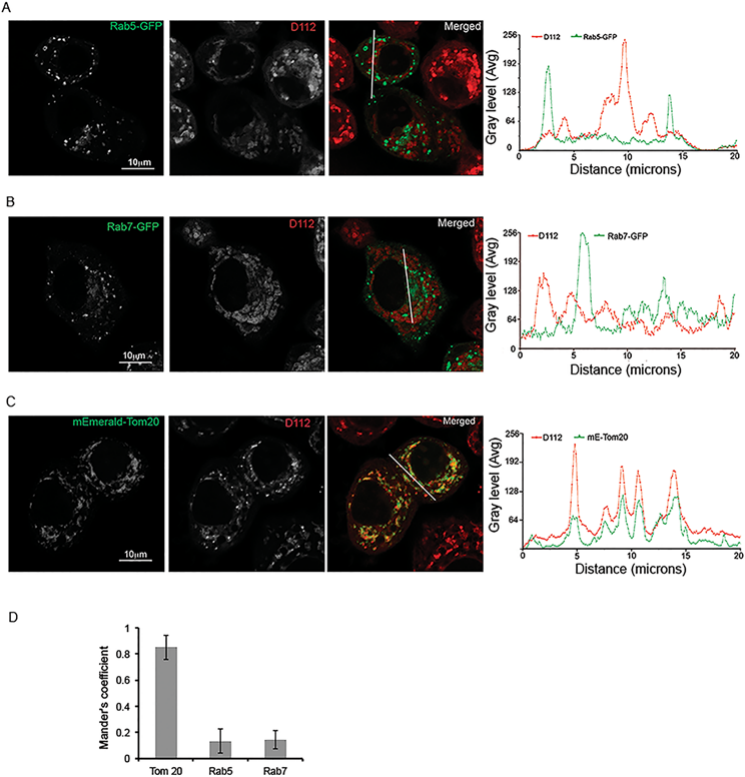
D112 co-localizes with mitochondria. SK-BR-3 cells were transiently transfected with the indicated plasmids expressing either mEmerald-Tom20, Rab5-GFP or Rab7-GF. Cells were treated with 0.25 μg/ml D112 for 4 h and then live imaging was performed with confocal microscopy. (A) D112 did not co-localize with Rab5. (B) D112 did not co-localize with Rab7. (C) D112 co-localized with Tom 20. The experiment was performed independently twice and representative images are shown. (D) Summary of the Mander’s correlation coefficients of Rab5, Rab7 or Tom20 and D112 in ten SK-BR-3 cells separately.

Given that D112 induced the mitochondrial apoptotic pathway, we wanted to explore the physical link between D112 and mitochondria. D112 has a positive charge delocalizing between two nitrogens that gives D112 a visible purple color. We determined that D112 fluoresces with an excitation wavelength of 540–561 nm and emission wavelength of 560–680 nm, with a peak at 620–630 nm. We thus took advantage of these properties to visualize D112 within the cell. Adherent SK-BR-3 breast cancer cells were used for these experiments as non-adherent Jurkat cells have minimal cytoplasmic space that is not ideal for morphological studies. We observed intracellular punctate staining of D112 in the cytoplasm (Fig 4). We attempted to use indirect immunofluorescence to co-localize D112 staining with organelle markers, however D112 fluorescence was lost upon cellular fixation and permeabilization. Therefore, we conducted live cell confocal imaging in cells expressing fluorescently tagged markers. We first asked whether D112 entered the cell via endocytosis, since endocytosis is a common pathway by which cells take up extracellular molecules [27]. To this aim, we examined the co-localization of D112 with two endosomal markers. The early endosome marker Rab5-GFP (Fig 4A) and late endosome marker Rab7-GFP (Fig 4B) were transiently transfected into SK-BR-3 cells, respectively. The transfected cells were incubated with D112 for 4 h, and live images were captured with a confocal microscope. We observed that D112 co-localized with neither Rab5 nor Rab7, suggesting that endocytosis was not responsible for D112 cellular uptake. Because endocytosis is an early event, we also recorded D112 localization at earlier time points (S6 and S7 Figs). Even at the 5-minute time point, D112 was not significantly localized with either endosomal marker. We next analyzed D112 localization with respect to the mitochondrial outer membrane protein mEmerald-Tom 20 (Fig 4C). We observed that the D112 signal strongly overlapped with the mitochondrial marker. Linescan analysis of each fluorescent channel indicated an association of D112 with mitochondria and this co-localization was confirmed as statistically significant by Mander’s correlation analysis (Fig 4D). Thus D112 associates with mitochondria and this proximity may contribute to the D112-induced activation of the mitochondrial apoptotic pathway.

### D112 shows increased toxicity to breast cancer cells and metastatic melanoma cells

**Fig 5:**
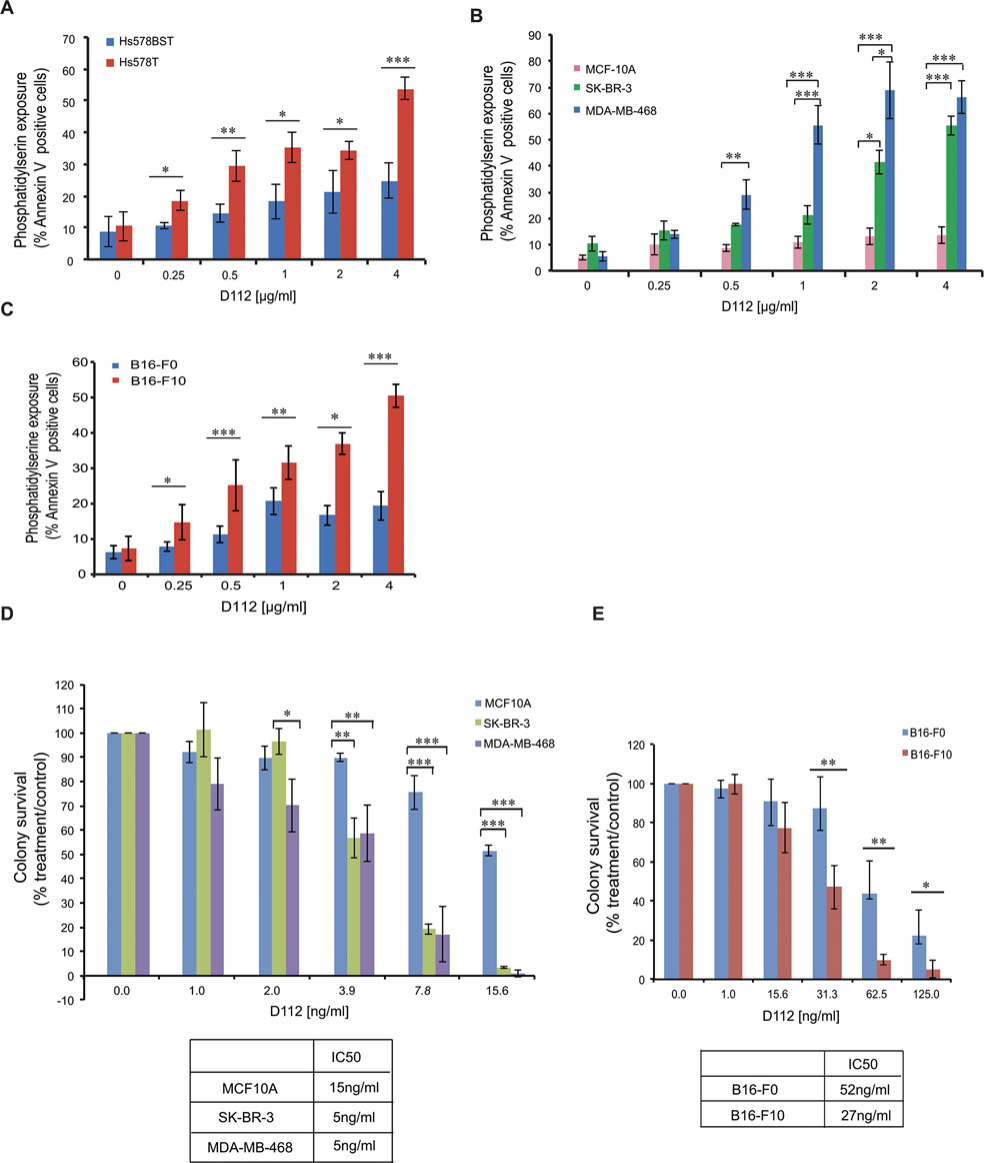
D112 shows selective cytotoxicity against transformed cells. (A) Paired human normal breast cell line Hs 578Bst and human breast carcinoma cell line Hs 578T. (B) Non-transformed human mammary epithelial cell lines MCF-10A in comparison to the mammary carcinoma cell lines SK-BR-3 and MDA-MB-468. (C) Melanoma parental cell line B16-F0 and melanoma metastatic cell line B16-F10. All cell lines were treated with the indicated amounts of D112 for 24 h and apoptosis was indicated by PS exposure, which was measured as described previously. (D) (E) Long-term colony formation of non-transformed cell lines and cancer cell lines. Cell lines were treated with the indicated amounts of D112 for 24 h, and all cells were harvested, counted and seeded in 24-well plates. Colonies were stained with crystal blue and counted after 7–14 days incubation. Mean ± SD of three independent experiments performed in triplicate are shown, *P<0.05, **P<0.01, ***P<0.001.

We first became interested in studying D112 based on the initial reports that D112 had selective toxicity to a human colon cancer cell line in comparison to a normal monkey kidney cell line. However, common anti-cancer treatments are less effective against solid tumors versus haematological malignancies (as exemplified by Jurkat cells) and show a non-apoptotic mechanism of action [28–30]. Thus we examined whether D112 induced apoptosis in cell lines derived from solid tumors and whether D112 showed any selectivity for transformed or metastatic cancer cells. Accordingly, we first analyzed the paired cell lines of Hs 578T and Hs 578Bst. Hs 578T is a human breast carcinoma cell line and Hs 578Bst is a normal cell line derived from non-transformed adjacent tissue from the same patient. The carcinoma cell line was significantly more sensitive to D112-induced apoptosis in comparison to the normal cell line (Fig 5A). We then expanded this observation to other breast cell lines (Fig 5B). We observed that the non-transformed human mammary epithelial cell line MCF-10A showed no significant dose-dependent response to D112 treatment. On the other hand, the mammary carcinoma lines, SK-BR-3 and MDA-MB-468, were significantly more sensitive to D112-induced apoptosis. We next took advantage of a progressive metastasis model that had been established in the mouse B16 cell lines [31]. The B16-F0 cell line is a mouse melanoma cell line, while B16-F10 is an aggressive metastatic cell line generated from the B16-F0 line through a ten-time *in vivo* selective procedure. The B16-F0 cells were more resistant to D112-induced apoptosis than the highly metastatic B16-F10 line (Fig 5C). Thus, in the cell lines that we tested, D112 triggered an increased apoptotic response in transformed versus normal cells and increased cytotoxicity to a metastatic versus a weakly metastatic line.

We next evaluated the effect of D112 on these adherent cell lines by the conventional clonogenic survival assay, which provides a read-out of the sum of cytotoxic (e.g. apoptosis) and cytostatic (growth arrest) responses [28, 29]. Cell lines were treated with D112 for 24 h and the number of clonal colonies (>50 cells) that formed over a span of 2 weeks was recorded (Fig 5D). MCF10A cells were three times more resistant to D112 treatment than SK-BR-3 or MDA-MB-468 cells, based on the IC50 of 15 versus 5 ng/ml. Additionally, weakly-metastatic B16-F0 showed a 2-fold greater resistance than the metastatic B16-F10 cell line (Fig 5E). Since this low dose of D112 was below the threshold to detect apoptosis, it appeared that, similar to Jneo cells, low doses of D112 induced proliferative arrest. Nevertheless, these assays suggest that both high and low doses of D112 have selective activity against the transformed/metastatic cell lines that we tested.

## Discussion

D112 was identified by Kodak as a cyanine dye that had higher toxicity against a cancer cell line than a non-transformed cell line. Whether this selectivity could be applied to other cancer cell lines was not known. Furthermore, the mechanism of D112-induced killing remained unclear. In this study, we investigated D112-mediated cell killing in a number of cell lines and identified that relatively high doses of D112 triggered the mitochondrial apoptotic pathway and lower doses induced cell cycle arrest.

To determine whether D112 induced apoptosis, we assessed the contribution and activation mechanism of caspase 3, the major effector caspase. We observed that D112 treatment produced active caspase 3 subunits p19/17. Incubation with caspase inhibitors prevented the generation of active caspase 3 subunits p19/17, and produced p20. It is known that caspase 3 is activated by two cleavage events. The first step of pro-caspase 3 activation, which gives rise to a p20 cleavage product, is mediated mostly by initiator caspases [32], such as caspase 9 [33]. Non-caspase proteases [34] and a wide range of stressors such as ER stress, IL-3-deprivation [35] or staurosporine [36] can also produce a p20 fragment that becomes apparent in the presence of zVAD-fmk. In many cases, the enzyme responsible for the generation of p20 is unclear, although in some cases chymotrypsin-like serine proteases can generate a p20 fragment [35]. Caspase 3 autocatalysis then produces the active subunits p19/17 [33]. As we observed that D112 produced similar caspase 3 proteolytic products, D112 likely stimulates an apoptotic pathway that is shared with other stress-inducers. Furthermore, we determined that elevated Bcl-2 levels inhibited not only caspase activity, but also the production of p20, indicating that Bcl-2 functioned upstream of both caspases and the initial caspase 3-protease. As Bcl-2 blocks mitochondrial dysfunction by inhibiting Bax/Bak pore formation, it is possible that the unidentified caspase 3-protease is localized to the mitochondria and is released to the cytosol through a Bax/Bak pore.

The second hallmark of apoptosis that we tested was mitochondrial depolarization. We observed that D112 induced mitochondrial depolarization was downstream of caspase activation. Mitochondrial membrane depolarization has been originally postulated to be an early event in the apoptotic pathway. However, more recent evidence suggests that it can also be a subsequent event in the apoptotic pathway, downstream of caspase activation. Mitochondrial potential was disrupted after caspase-3-mediated cleavage of protein kinase C delta (PKCδ), which allowed it to translocate to the mitochondria [37]. In another study, Fas-induced loss of mitochondrial potential occurred after the activation of caspase-1-like caspases, and the addition of caspase inhibitors inhibited mitochondrial membrane depolarization [38, 39]. Similarly, our study showed that the caspase pan-inhibitor z-VAD-fmk inhibited mitochondrial membrane depolarization, suggesting that mitochondrial depolarization was a downstream event in our case.

It is noteworthy that mitochondrial membrane depolarization was rescued by caspase inhibitors, whereas PS externalization was only partly inhibited in D112 induced cell death. For example, in the case of cells treated with 1 μg/ml D112, 20% more Annexin V-positive cells were detected under this condition even in the presence of zVAD-fmk. Conceivably, other non-caspase-dependent factors, for example Apoptosis-Inducing-Factor (AIF), and/or ATP-depletion may contribute to PS externalization [8]. In support of this, both caspase- and AIF- mediated PS exposures are dependent on mitochondrial dysfunction and are blocked by Bcl-2 [18]. Also, PS exposure is proposed to be a consequence of ATP depletion [40]. In healthy cells, ATP-dependent translocases maintain PS on the inside of the cell. During apoptosis, ATP levels decrease and translocase activity diminishes, leading to PS externalization [41]. In our study, D112 co-localized with mitochondria, and it is possible that D112 inhibits ATP production by interfering with mitochondrial respiration, hence contributing to PS exposure in a caspase-independent manner.

Finally, we observed that D112 displayed enhanced cytotoxicity towards transformed Hs 578T, SK-BR-3 and MDA-MB-468 breast carcinoma cell lines versus non-transformed Hs 578Bst and MCF-10A normal breast epithelial cell lines. Furthermore, D112 was more cytotoxic to the metastatic melanoma cell line B16-F10 versus the lesser aggressive parental B16-F0 cells. These data suggest that D112 may have potential to be tested as a selective anti-cancer compound that conceivably will cause less off-target toxic side effects. However, the selectivity that we observed was much reduced in comparison to the original study that identified a 900-fold difference in sensitivity between a human colon cancer cell line and non-transformed monkey kidney cell line [15]. This is likely because of the difference in cell lines and species differences in the original study. In our current study, we compared cell lines from the same species, of which, one set was originally isolated from tissue from the same patient (Hs578T vs 578 Bst) whereas another set was clonally derived from each other (B16F0 vs B16F10). The difference in sensitivity that we observed between our cell lines was only 2- and 3-fold. At this time, we are not able to predict whether this difference in sensitivity could translate into a selective anticancer agent.

Our current work demonstrated that D112 localizes to mitochondria and induces Bax/Bak-dependent mitochondrial outer membrane permeabilization, cytochrome c release and activation of caspases 9 and 3. Mitochondrial depolarization was dependent on caspase activation, suggesting a positive amplification loop for mitochondrial dysfunction. We speculate that this loop may involve activity of caspase-cleaved Bid and is unlikely to require Permeability Transition Pore, since preliminary studies with cyclosporin A that blocks PTP activation did not decrease apoptosis. With respect to upstream signaling pathways, it is not clear how D112 triggers mitochondrial effects. Perhaps, due to its redox potential, D112 may inhibit the electron transport chain and elevate reactive oxygen species that trigger mitochondrial damage and apoptosis. Further studies will address the mechanism of increased toxicity of the transformed versus non-transformed cell lines that we have shown here. These future studies will determine whether D112 may serve as a prototype for optimization of novel molecules with even more enhanced selective activity.

## Materials and Methods

### Cell culture and chemicals

Stable transfected human T-cell leukemia Jurkat cell lines Jneo and JBcl2 were kindly provided by Dr. Chris Bleackley (University of Alberta), JR cells were kindly provided by Dr. Hannah Rabinowich (University of Pittsburgh). The human normal breast epithelial cell lines MCF-10A was obtained from ATCC and maintained as previously described [42]. B16-F0 (ATCC, CRL-6322), B16-F10 (ATCC, CRL-6475), Hs578BST (ATCC, HTB-125) and Hs578T (ATCC, HTB-126) cells were obtained from ATCC (Manassas, VA, USA). B16-F0, B16-F10 and Hs578T were maintained in Dulbecco's Modified Eagle's Medium, high glucose, with 10% FCS; Hs578BST was maintained in Dulbecco's Modified Eagle's Medium, high glucose, with 10% FCS and 30ng/ml EGF. Z-VAD-fmk and z-DEVD-fmk were purchased from BD Pharmingen (Mississauga, ON, Canada), SYTOX Green death stain and Alexa Fluor 647 Annexin V conjugate were all obtained from Invitrogen (Carlsbad, CA, USA). All chemicals were purchased from Sigma-Aldrich (St Louis, MO, USA) unless indicated otherwise.

### Western blotting

Jurkat cells were treated with 0, 0.25, 0.5, 1, 2 or 4 μg/ml D112 for 24 h. All cells were harvested and lysed in lysis buffer (50 mM Tris pH 8, 150 mM NaCl, 1% NP-40, 0.5% sodium deoxycholate, 1mM β-mercaptoethanol). The whole cell lysates were then mixed with 6X SDS-PAGE gel loading buffer and boiled for 10 min. Protein concentration was measured using Pierce BCA protein assay kit from Thermo Fisher Scientific Inc (Waltham, MA). 15 μg proteins from cell lysates were loaded onto 14% SDS polyacrylamide gels and subjected to SDS-PAGE analysis. Proteins were resolved at 100 V and subsequently transferred to PVDF membranes by use of Bio-Rad Mini-Gel Box Electrotransfer for 1 h at 300 mA. Membranes were blocked in phosphate-buffered saline (PBS) containing 0.1% Tween 20 and 5% skimmed milk for 1 h. Proteins were visualized with specific primary antibodies, followed by goat anti—mouse or rabbit HRP-conjugated secondary antibodies.

Antibody against caspase 3 (ADI-AAP-13) was from Enzo Life Science (Farmingdale, NY, USA); Caspase 9 antibody (9502), Bax antibody (2774) and Bcl-2 (2872) antibody were from Cell Signaling (Boston, MA, USA); Bak antibody (06–536) was from Thermo Fisher Scientific; Tom-20 antibody (SC-11415) was from Santa Cruz (Dallas, TX); Cytochrome c antibody (556433) was from BD Pharmingen (San Diego, CA) and antibody against Tubulin (T5168) was from Sigma-Aldrich.

### Determination of apoptosis

Apoptosis was determined by analyzing phosphatidylserine exposure using Alexa Fluor 647 Annexin V conjugate staining and mitochondria dysfunction indicated by loss of DiOC_6_ fluorescence. Briefly, cells were washed 3 times with PBS after D112 treatment. For Annexin V 647 staining, cells were resuspended in 100 μL annexin-binding buffer (10 mM HEPES, 140 mM NaCl, and 2.5 mM CaCl_2_, pH 7.4) with 2.5 μL Alexa Fluro 647 Annexin V conjugate, and incubated for 15 min at room temperature. After washing with PBS, the percentage of cells binding Alexa Fluor 647 Annexin V was determined using an Accuri C6 flow cytometer (BD Accuri, Ann Arbor, MI, USA) in the FL-4 channel. For DiOC_6_ staining, cells were stained with 40 ng/ml DiOC_6_ in PBS for 10 min at room temperature, and loss of electrochemical potential was determined by loss of fluorescence in the FL-1 channel as measured by flow cytometry. D112 was measured in the FL-2 channel.

### Caspase 3 enzymatic activation assay

2x10^7^ cells were treated with D112 at indicated concentrations for 24 h. All cells were harvested and washed twice with PBS, then resuspended in 100 μl lysis buffer (50 mM HEPES, 100 mM NaCl, 0.1% CHAPS, 1 mM DTT, 100 mM EDTA, pH 7.4) on ice for 20 min. Protein concentration was measured using a Pierce BCA protein assay kit. 100 μg protein from each assay was diluted in 50 μl cell lysis buffer and mixed with 50 μl of 2X Reaction Buffer (50 mM HEPES, 100 mM NaCl, 0.1% CHAPS, 10 mM DTT, 100 mM EDTA, 10% glycerol, pH 7.4). 5 μl of the 4 mM DEVD-AFC substrate (200 μM final concentration) was added to the mixture and incubated at 37°C for 1 h. Caspase 3 activity was determined at 405 nm in a 96-well plate on a Multiskan Ascent plate reader (Thermo Fisher Scientific Inc).

### Detection of necrosis

Cell death was detected by SYTOX Green death stain. Briefly, cells were harvested after treatment, and resuspended in binding buffer containing 1 μl/ml of SYTOX Green stain solution. Next, cells were incubated for 20 min at room temperature before analysis. Signals were collected in in the FL-1 channel as measured by flow cytometry.

### Cell viability assay

Cell viability was determined by trypan blue exclusion method. Briefly, Jneo, JBcl-2 and JR cells were treated with D112, and then cells were harvested and stained with 0.4% trypan blue solution. The number of blue staining cells and total cells were counted, and cell survival rate was calculated.

### Mitochondria fractionation

Mitochondrial fractionation was conducted as described previously [34]. Jneo cells were treated with D112 or staurosporine (as a positive control) at indicated concentrations for 24 h. 2 × 10^6^ cells per sample were harvested and cell pellets was then re-suspended in 100 μl of digitonin lysis buffer (75 mM NaCl, 1 mM NaH_2_PO_4_, 8 mM Na_2_HPO_4_, 250 mM sucrose, 1% digitonin) and incubated on ice for 10 min. Lysate was then centrifuged for 5 min at 14,000 rpm. Supernatant (cytosolic fraction) was transferred to another tube and pellet containing heavy membrane, including mitochondria, was re-suspended in 200 μl of Triton X-100 lysis buffer (0.1% Triton X-100, 25 mM Tris pH 8.0). Both supernatant and pellet were mixed with SDS loading buffer and subjected to western blotting.

### Transient transfection

SK-BR-3 cells were seeded in an 8-well Nunc Lab-Tek chambered coverglass (Sigma) and reached 70% confluence for transfection. To form the transfection complex, 10 μl pre-warmed Opti-MEMI Reduced-Serum Medium (Life Technologies, Burlington, ON, Canada) with 0.2 μg plasmid DNA was mixed with an equal volume of Opti-MEMI Reduced-Serum Medium with 0.4 μl TransIT-LT1 reagent (Mirus, Madison, WI, USA). After 20 min incubation at room temperature, the complex was added dropwise to different areas of the wells and the cells were incubated for 6 h for delivery. Complex was then replaced by fresh complete growth medium for gene expression and membrane recovery before the next treatment. Plasmid mEmerald-Tom20 was kindly supplied by Dr. Robert E. Campbell (Department of Chemistry, University of Alberta, Canada).

### Confocal imaging of living cells

For confocal live cell microscopy, SK-BR-3 cells transfected with plasmids were treated with 0.25 μg /ml D112 for 4 h. Images were then directly taken on a Zeiss LSM 710 inverted confocal microscope fitted with a 40x 1.4 NA Oil DIC Plan-Apochromat objective (Carl Zeiss Canada, Toronto, ON, Canada). After acquisition, images were visualized using ZEN 2009 Light Edition (Carl Zeiss) and MetaXpress (Version 5.2) (Molecular Devices) software.

### Image analysis

Image analysis and processing was performed in MetaXpress and Fiji (online open-source, http://fiji.sc/Fiji). Linescan analysis was performed on MetaXpress. Calculation of Mander’s correlation coefficient for colocalization analysis was performed on Fiji. In brief, a region of interest (ROI) was applied to transfected cells. A manual threshold was applied to each channel using ‘dark background and the Mander’s correlation calculation was run by Coloc 2 method of Fiji software (http://fiji.sc/Colocalization_Analysis#Colocalization_analysis_using_Coloc_2).

### Cell proliferation

Jneo, JBcl-2, JR cells were treated with 62.5ng/ml D112 for 24 h. All cells were washed and re-suspended in fresh medium. 10^4^ cells (per well) were seeded in 24-well plates. Cell number was counted every 24 h.

### Colony formation

Multiple cell lines were treated with indicated doses of D112 for 24 h. All cells were harvested and seeded in 12-well plates for colony formation. Considering non-transformed cell lines showed lower colony forming ability than cancer cell lines under normal conditions, for MCF-10A cell line, 500 cells were seeded, while for SK-BR-3, MDA-MB-468, B16-F0 and B16-F10 cell lines, 100 cells were seeded. Cells were incubated for 7–14 days. Colonies were then fixed and stained with crystal violet solution with 0.05% (w/v) crystal violet, 4% formaldehyde in 1 X PBS.

### Statistical analysis

Data are presented as a mean of three independent experiments, with error bars indicating the SD. Statistical significance was determined using a two-tailed Student’s t-test for two means with equal variance. For statistical analysis of multiple groups (Fig 5B and 5D), the one-way Analysis of Variance (*ANOVA*) test was performed and p-values were obtained by Tukey’s Post Hoc test.

## Supporting Information

S1 Fig D112 induced apoptosis and secondary necrosis. Jneo cells were treated with 2 μg/ml of D112 for the indicated time points and then double labeled with SYTOX green and Alexa Fluro 647 Annexin V. Cells were analyzed by flow cytometry. Shown is a representative of one of three experiments performed in triplicate.
(TIFF)

S2 Fig D112 induced apoptosis and secondary necrosis. Jneo cells were treated with 2 μg/ml of D112 in the presence of zVAD-fmk (20 μM) for the indicated time points and then double labeled with SYTOX green and Alexa Fluro 647 Annexin V. All cells were analyzed by flow cytometry. Shown is a representative of one of three experiments performed in triplicate.
(TIFF)

S3 Fig BH3-only proteins were examined by western blotting. Jneo cells were incubated for 24 h with increasing concentrations of D112 as indicated. Whole cell lysates were subjected to western blotting analysis with the indicated antibodies. The experiment was performed independently three times and a representative blot is shown. Bid (2002), Puma (12450) and Bim (2819) antibodies were from Cell Signaling; Bik (sc1710) antibody was from Santa Cruz; Noxa (ab13654) antibody was from Abcam.
(EPS)

S4 Fig Permeability Transition Pore was not involved in D112-induced cell death. Jneo cells were treated with the indicated concentrations of D112 in the presence or absence of 5 μM cyclosporine A (CsA) for 24 h. Jneo cells were treated with H_2_O_2_ (200 μM and 400 μM) for 4 hours, as a positive control [43]. Phosphatidylserine exposure indicated by Alexa Fluor 647-annexin V positive cells is shown as a percent of total cells as determined by flow cytometry. In all experiments, the mean ± SD of three independent experiments performed in triplicate are shown, *P<0.05, **P<0.01, ***P<0.001.
(EPS)

S5 Fig The extrinsic pathway did not contribute to D112-induced apoptosis. (A) caspase 8 cleavage was examined by western blotting. Cleaved caspase 8 antibody (9496) was from cell signaling. (B) Jneo and Spi-2 expressing Jurkat cells were treated with the indicated concentrations of D112 for 24 h. Phosphatidylserine exposure indicated by Alexa Fluor 647-annexin V positive cells is shown as a percent of total cells as determined by flow cytometry. In all experiments, the mean ± SD of three independent experiments performed in triplicate are shown, *P<0.05, **P<0.01, ***P<0.001.
(EPS)

S6 Fig Endocytosis was not involved in D112 intracellular uptake. SK-BR-3 cells were transiently transfected with Rab5-GFP. Cells were treated with 0.25 μg/ml D112 for 5 min, 15 min, 30 min or 60 min, and then live imaging was performed with confocal microscopy. Summary of the Mander’s correlation coefficients of Rab5 and D112 in ten SK-BR-3 cells was showed at the bottom. Mean ± SD of three independent experiments performed in triplicate are shown.
(TIF)

S7 Fig Endocytosis was not involved in D112 intracellular uptake. SK-BR-3 cells were transiently transfected with Rab7-GFP. Cells were treated with 0.25 μg/ml D112 for 5 min, 15 min, 30 min or 60 min, and then live imaging was performed with confocal microscopy. Summary of the Mander’s correlation coefficients of Rab7 and D112 in ten SK-BR-3 cells was showed at the bottom. Mean ± SD of three independent experiments performed in triplicate are shown.
(TIF)
