# Supplementary figures and images for: Characterization of the Apoptotic Response Induced by the Cyanine Dye D112: A Potentially Selective Anti-Cancer Compound
Source: PLoS One. 2015 Apr 30;10(4):e0125381. doi: 10.1371/journal.pone.0125381 (PMC4415924; doi:10.1371/journal.pone.0125381)

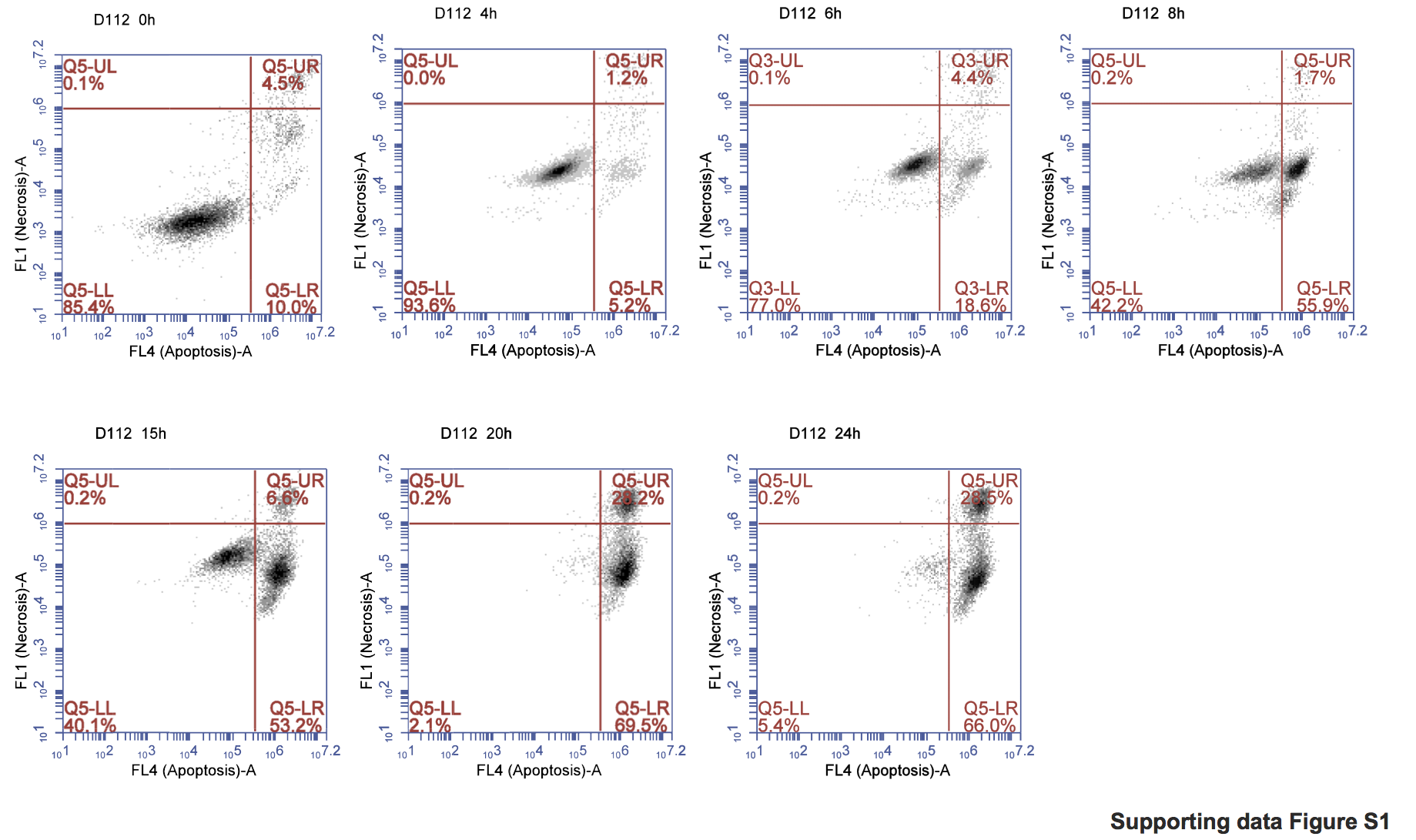

Supplement: S1 Fig — Jneo cells were treated with 2 μg/ml of D112 for the indicated time points and then double labeled with SYTOX green and Alexa Fluro 647 Annexin V. Cells were analyzed by flow cytometry. Shown is a representative of one of three experiments performed in triplicate. (TIFF) [file pone.0125381.s001.tiff]

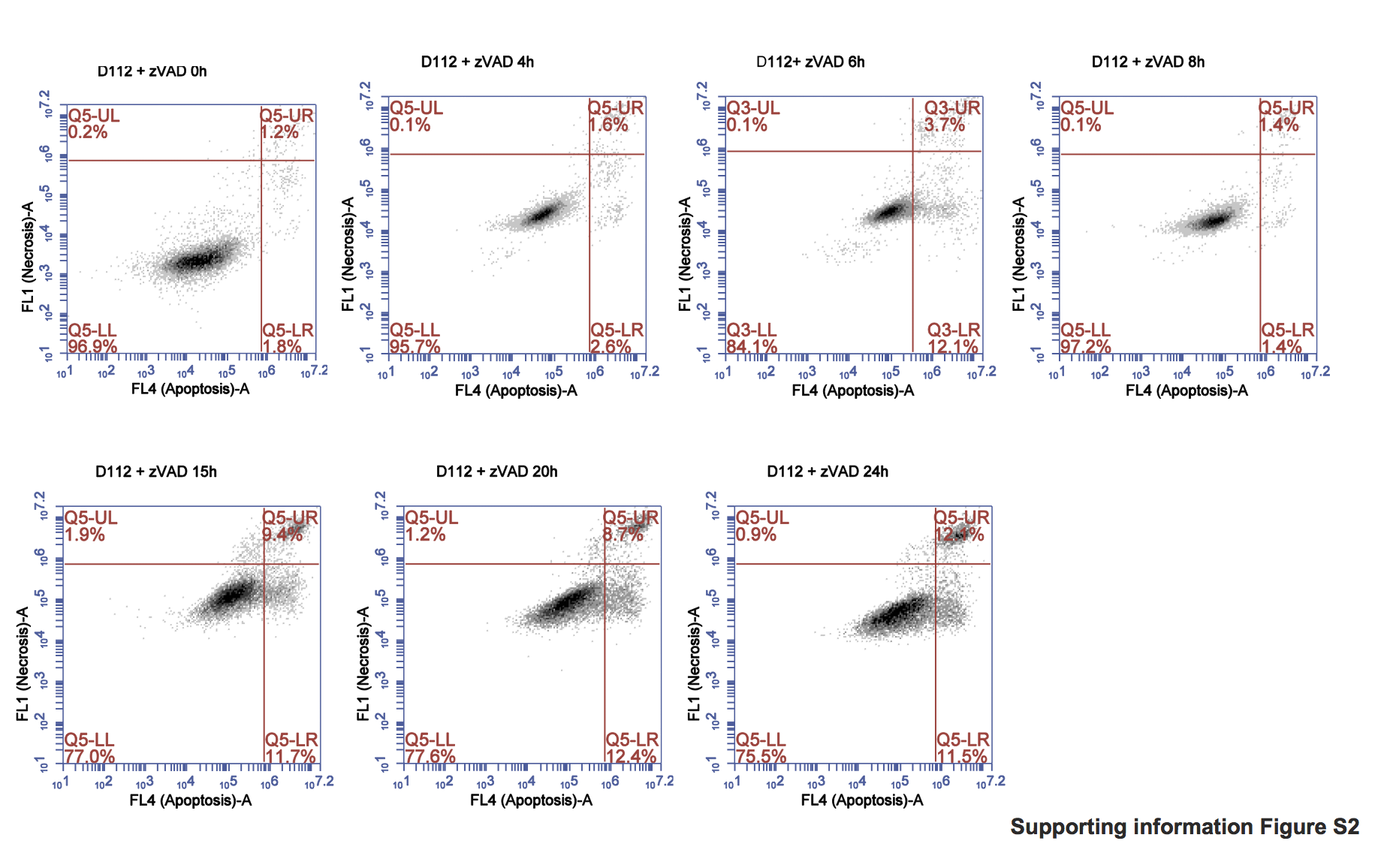

Supplement: S2 Fig — Jneo cells were treated with 2 μg/ml of D112 in the presence of zVAD-fmk (20 μM) for the indicated time points and then double labeled with SYTOX green and Alexa Fluro 647 Annexin V. All cells were analyzed by flow cytometry. Shown is a representative of one of three experiments performed in triplicate. (TIFF) [file pone.0125381.s002.tiff]

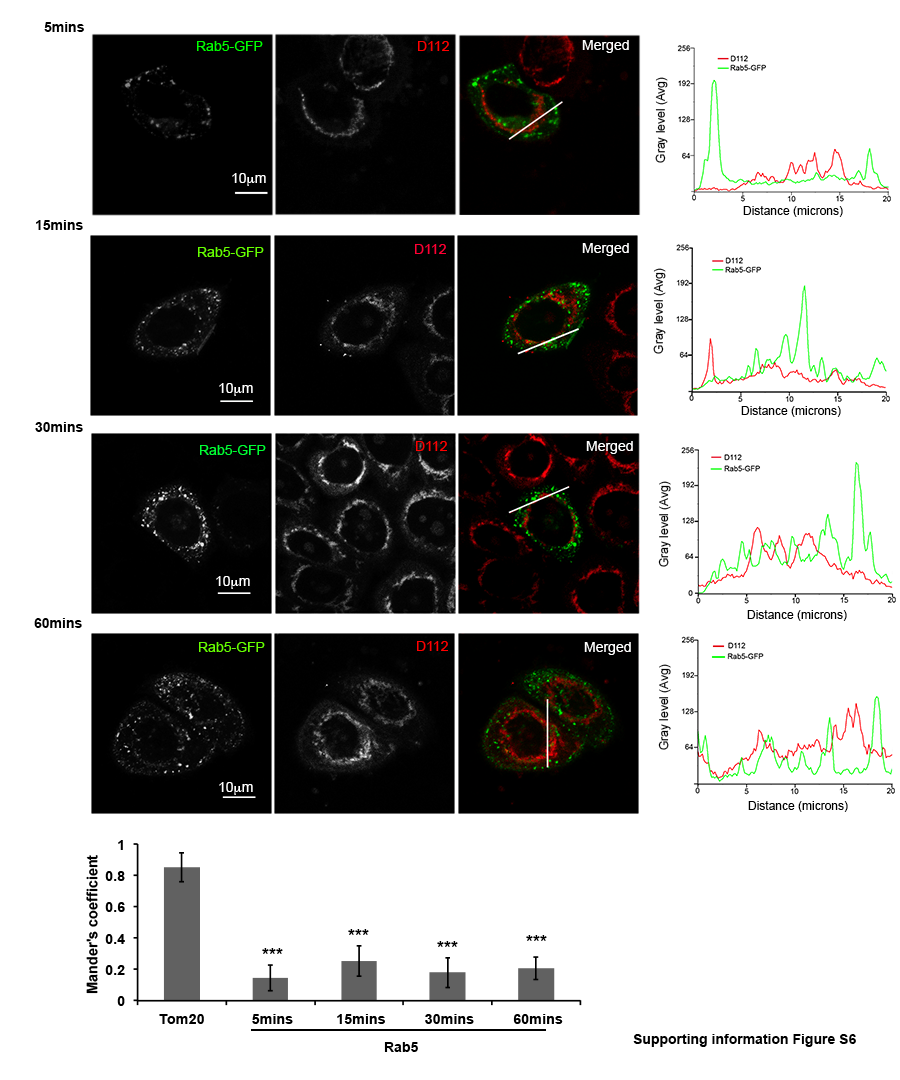

Supplement: S6 Fig — SK-BR-3 cells were transiently transfected with Rab5-GFP. Cells were treated with 0.25 μg/ml D112 for 5 min, 15 min, 30 min or 60 min, and then live imaging was performed with confocal microscopy. Summary of the Mander’s correlation coefficients of Rab5 and D112 in ten SK-BR-3 cells was showed at the bottom. Mean ± SD of three independent experiments performed in triplicate are shown. (TIF) [file pone.0125381.s006.tif]

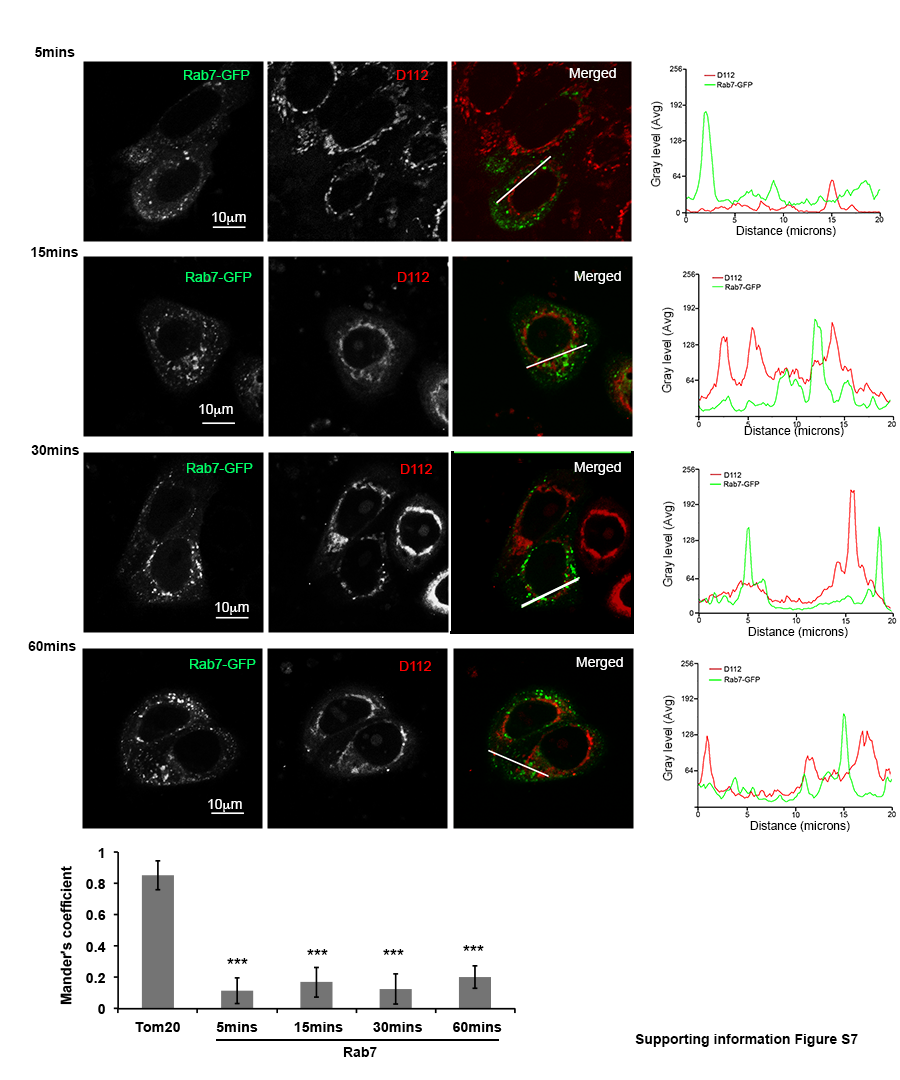

Supplement: S7 Fig — SK-BR-3 cells were transiently transfected with Rab7-GFP. Cells were treated with 0.25 μg/ml D112 for 5 min, 15 min, 30 min or 60 min, and then live imaging was performed with confocal microscopy. Summary of the Mander’s correlation coefficients of Rab7 and D112 in ten SK-BR-3 cells was showed at the bottom. Mean ± SD of three independent experiments performed in triplicate are shown. (TIF) [file pone.0125381.s007.tif]
